# Insights into the mechanism of *Arnebia euchroma* on leukemia via network pharmacology approach

**DOI:** 10.1186/s12906-020-03106-z

**Published:** 2020-10-27

**Authors:** Biting Wang, Zengrui Wu, Jiye Wang, Weihua Li, Guixia Liu, Bo Zhang, Yun Tang

**Affiliations:** 1 Shanghai Key Laboratory of New Drug Design, School of Pharmacy, East China University of Science and Technology, Shanghai, 200237 China; 2 Key Laboratory of Xinjiang Phytomedicine Resource and Utilization, Ministry of Education, School of Pharmacy, Shihezi University, Shihezi, 832002 China

**Keywords:** *Arnebia euchroma*, Concentration, Leukemia, Traditional Chinese medicine, Network pharmacology, Weighted network

## Abstract

**Background:**

*Arnebia euchroma (A. euchroma)* is a traditional Chinese medicine (TCM) used for the treatment of blood diseases including leukemia. In recent years, many studies have been conducted on the anti-tumor effect of shikonin and its derivatives, the major active components of *A. euchroma*. However, the underlying mechanism of action (MoA) for all the components of *A. euchroma* on leukemia has not been explored systematically.

**Methods:**

In this study, we analyzed the MoA of *A. euchroma* on leukemia via network pharmacology approach. Firstly, the chemical components and their concentrations in *A. euchroma* as well as leukemia-related targets were collected. Next, we predicted compound-target interactions (CTIs) with our balanced substructure-drug-target network-based inference (bSDTNBI) method. The known and predicted targets of *A. euchroma* and leukemia-related targets were merged together to construct *A. euchroma*-leukemia protein-protein interactions (PPIs) network. Then, weighted compound-target bipartite network was constructed according to combination of eight central attributes with concentration information through Cytoscape. Additionally, molecular docking simulation was performed to calculate whether the components and predicted targets have interactions or not.

**Results:**

A total of 65 components of *A. euchroma* were obtained and 27 of them with concentration information, which were involved in 157 targets and 779 compound-target interactions (CTIs). Following the calculation of eight central attributes of targets in *A. euchroma*-leukemia PPI network, 37 targets with all central attributes greater than the median values were selected to construct the weighted compound-target bipartite network and do the KEGG pathway analysis. We found that *A. euchroma* candidate targets were significantly associated with several apoptosis and inflammation-related biological pathways, such as MAPK signaling, PI3K-Akt signaling, IL-17 signaling, and T cell receptor signaling pathways. Moreover, molecular docking simulation demonstrated that there were eight pairs of predicted CTIs had the strong binding free energy.

**Conclusions:**

This study deciphered that the efficacy of *A. euchroma* in the treatment of leukemia might be attributed to 10 targets and 14 components, which were associated with inhibiting leukemia cell survival and inducing apoptosis, relieving inflammatory environment and inhibiting angiogenesis.

## Background

Leukemia is a common malignancy in children and adults originating from pluripotent hematopoietic stem cells. Due to various reasons, leukemia cells undergo enhanced self-renewal, over proliferation, and blocked differentiation and apoptosis. Abnormal immature cells proliferate and accumulate in bone marrow and other hematopoietic tissues, leading to the suppression of normal hematopoiesis and infiltration of other organs and tissues. According to the types of cells affected and the developmental stage of the originating cells, leukemia is classified into four major categories: acute myeloid leukemia (AML), acute lymphoblastic leukemia (ALL), chronic myeloid leukemia (CML), and chronic lymphoblastic leukemia (CLL).

The usual approach to treat leukemia is the use of toxic compounds to kill cancer cells, which will eventually destroy the immune system, and make patients susceptible to fatal bacterial and fungal infections. For that reason, it was suggested that approaches such as inducing leukemia cell differentiation and apoptosis other than killing cells may be more effective in the treatment of leukemia [1]. Traditional Chinese medicine (TCM) has shown potentials for the treatment of leukemia, for example, arsenic trioxide (ATO), the main component of TCM white arsenic, has been applied in the treatment of leukemia [2].

*Arnebia euchroma* (Royle) Jonst., also called Xinjiang Zicao, is a TCM used in the treatment of blood diseases including leukemia [3]. It has been reported that among the 295 empirical prescriptions for treating haematological diseases in Chinese medicine, the frequency of *A. euchroma* is 10.3%, and the frequency of *A. euchroma* powder, a prescription that takes *A. euchroma* as the monarch herb is 53.1% [4]. The components of *A. euchroma* consist of two major categories: one is hydrophilic components, mainly a mixture of polysaccharides and glycoproteins; the other is lipophilic substances with a variety of biological activities, including naphthoquinones, alkaloids, monoterpene phenol and benzoquinones, organic acid ester, and so on. Among these, typical naphthoquinones include shikonin and a series of derivatives [5], which have a common scaffold of 5,8-dihydroxy-2-isohexene-1,4-naphthoquinone. According to the optical configuration of the chiral center in the side chain, naphthoquinones can be divided into two subtypes: *R*-configuration (named shikonin) and *S*-configuration (named alkannin) (see Supplementary Figure S1) [6]. Studies have shown that naphthoquinones are the major active ingredients of *A. euchroma* that exert its pharmacological effects, whose concentrations are no more than 7% [7, 8]. Especially, some studies demonstrated that shikonin has therapeutic effects on leukemia cells, mainly including HL-60 cells and K562 cells [9–11]. However, the underlying mechanism of action (MoA) for all the components of *A. euchroma* on leukemia is barely explored systematically.

In 2007, network pharmacology was proposed to describe the multiple interactions among drugs, targets and diseases [12, 13], which coincides with the “multi-component, multi-target” characteristics of TCM and has been widely used in the research and development of TCM. In this field, our group has developed a series of computational methods to predict compound-target interactions (CTIs), including network-based inference (NBI), substructure-drug-target network-based inference (SDTNBI), and balanced SDTNBI (bSDTNBI) [14–16]. In previous studies we have demonstrated that these methods could predict potential targets for TCM components and help to understand MoA of TCM reasonably [17–19].

Since NBI only can predict targets for old compounds within a known CTI network, while bSDTNBI has demonstrated better performance than SDTNBI in prediction of targets for new compounds outside the known CTI network in our previous publications. Therefore, in this study, we used bSDTNBI to predict targets for components of *A. euchroma*, and then investigated the material basis and MoA of *A. euchroma* on leukemia via network pharmacology approach, which may provide some basis and enlightenment to deeply explore the chemical and pharmacological basis of TCM.

## Methods

### Data collection and preparation

The components and their concentrations in *A. euchroma* were collected from several sources including TCM-MESH (http://mesh.tcm.microbioinformatics.org/, entering at Apr 2019) [20], TCMID (http://119.3.41.228/tcmid/, entering at Apr 2019) [21], Chinese Natural Product Chemical Composition Library (http://pharmdata.ncmi.cn/cnpc/, entering at Apr 2019), and Phytochemicals database (http://chemdb.sgst.cn/scdb/main/plant_introduce.asp, entering at Apr 2019), as well as related literature from PubMed (https://www.ncbi.nlm.nih.gov/pubmed/, entering at Apr 2019), Web of Science (http://apps.webofknowledge.com, entering at Apr 2019) and CNKI (http://www.cnki.net/, entering at Apr 2019). The structures of these compounds were obtained from NCBI PubChem Compound Database (https://www.ncbi.nlm.nih.gov/pccompound, entering at Apr 2019). Duplicates were removed.

Known compound-target interactions (KCTIs) for each component of *A. euchroma* were collected from four databases, including BindingDB (http://www.bindingdb.org/bind/index.jsp, entering at Apr 2019) [22], IUPHAR/BPS Guide to PHARMACOLOGY (https://www.guidetopharmacology.org/, entering at Apr 2019) [23], ChEMBL (https://www.ebi.ac.uk/chembl/, entering at Apr 2019) [24], and PubChem (https://pubchem.ncbi.nlm.nih.gov/, entering at Apr 2019) [25]. Target protein names of components were converted into gene names with the species limited into “*Homo sapiens*” by UniProt (https://www.uniprot.org/, entering at Apr 2019). Duplicated KCTIs were removed.

Leukemia related targets were obtained from five databases: OMIM (https://www.omim.org/, updated on Jan. 3, 2018) [26], PharmGkb (https://www.pharmgkb.org/, updated on Dec. 28, 2017) [27], TTD (http://bidd.nus.edu.sg/group/cjttd/, updated on Sep. 15, 2017) [28], DisGeNET (https://www.disgenet.org/search, entering at Apr 2019) [29], and MalaCards (https://www.malacards.org/, entering at Apr 2019) [30].

### Prediction of compound-target interactions

The bSDTNBI method was used to predict potential compound-target interactions (PCTIs) for each component of *A. euchroma*. In bSDTNBI method, three parameters, namely α, β and γ, should be determined first. Among them, α controls the relative importance of substructure vs. target nodes, and β defines the importance of drug-substructure vs. drug-target edges, while γ determines the importance of hub nodes. The three parameters were optimized by grid search and 10-fold cross validation. First of all, α and β were optimized under the condition of ignoring the influence of hub nodes (γ = 0). Then, under the optimal values of α and β, the optimal value of parameter γ was searched. Therefore, with the optimal parameters α, β and γ, bSDTNBI method was used to predict 10 potential targets for each component of *A. euchroma*. The predicted targets were further standardized as official gene symbols. Eventually, the collected known and predicted CTIs were integrated.

### Construction of PPI network and selection of leukemia-related targets

Targets of components of *A. euchroma* (T_A_) and targets related to leukemia (T_L_) were imported into Cytoscape 3.6.0, respectively. Protein-protein interactions (PPIs) of T_A_ and T_L_ were obtained by stringAPP, a Cytoscape plugin. High-confidence PPIs with scores above 0.7 were selected to construct *A. euchroma* PPI network (PPI_A_) and leukemia PPI network (PPI_L_). Then PPI_A_ and PPI_L_ were merged together to construct *A. euchroma*-leukemia PPI network.

*A. euchroma*-leukemia PPI network was analyzed by CytoNCA, another Cytoscape plugin. Each node in the network has eight central attributes, including subgraph centrality (SC), degree centrality (DC), eigenvector centrality (EC), information centrality (IC), local average connectivity-based method (LAC), betweenness centrality (BC), closeness centrality (CC), and network centrality (NC). For all target nodes, the targets of T_A_ that meet the screening criteria “SC > median SC & DC > median DC & EC > median EC & IC > median IC & LAC > median LAC & BC > median BC & CC > median CC & NC > median NC” were regarded as leukemia-related targets of *A. euchroma* (T_AL_) and were retained [31].

### Construction of weighted compound-target bipartite network

Compound-target interactions for T_AL_ were obtained from all CTIs (KCTIs + PCTIs). Here the concentrations of components in *A. euchroma* were used to weight the importance of each component. Since the concentrations of components were determined under different experimental conditions and influenced by many factors, such as the origin and growth time of *A. euchroma*, we processed the original concentration data as follows. Firstly, all the concentrations of components were summarized and standardized to a uniform percentage (mass percentage). Then, the average value of percentage of each component was calculated after the maximum and minimum values were removed. Finally, with “percent concentration value = 0.1%” as the threshold, components were divided into three classes, and the formula was as following:

$$ Components\left\{\begin{array}{c} Class\ I: weight=3,\kern0.5em concentration>0.1\%\\ {} Class\  II:\kern0.5em weight=2,\kern0.5em 0< concentration<0.1\%\\ {} Class\  II I: weight=1,\kern0.75em without\ concentration\ data\end{array}\right\} $$

After all components were weighted, a weighted compound-target bipartite network was constructed through Cytoscape.

### Enrichment of KEGG pathways

KEGG pathway enrichment analysis is usually used to describe the characteristics of query targets. Here, STRING 10.5 (https://string-db.org/cgi/input.pl, entering at Apr 2019) [32] was used to perform KEGG pathway enrichment analysis. The KEGG pathways with FDR (False Discovery Rate) < 0.05 were regarded as significant and useful. Targets in weighted compound-target bipartite network were inputted to STRING, and KEGG pathways with FDR < 0.05 were obtained. A scoring function was designed to screen pathways, and the equation was as following:

$$ Norm(Y)=\left(1-0.1\right)\ast \frac{Y_i-\mathit{\operatorname{MIN}}(Y)}{\mathit{\operatorname{MAX}}(Y)-\mathit{\operatorname{MIN}}(Y)}+0.1 $$

$$ {PS}_m= Norm\left(\sum \limits_{j=1}^{j={N}_T} Norm\left(\sum \limits_{i=1}^{i={N}_C}{X}_i\right)\right)+ Norm\left({LFDR}_m\right) $$

in which, *PS*_*m*_ refers to the score of the m-th KEGG pathway; *N*_*T*_ refers to the number of targets involved in the m-th pathway; *N*_*C*_ refers to the number of components that interact with the j-th target in the compound-target bipartite network; *X*_*i*_ refers to the weight of the i-th component; LFDR_m_ refers to the negative logarithmic value of FDR of the m-th pathway; *Norm* refers to the min-max normalization method with normalize data from 0.1 to 1.

After all pathways were scored, pathways that meet the role “score > 1.5 median score” were selected. Referring to the pathway classification standard of KEGG Pathway Database, here, these selected pathways were divided into four categories, including cell survival and death, immune system, endocrine system and specific human diseases. In order to facilitate analysis, we removed specific human disease pathways, which were not directly related to the treatment mechanism of leukemia, and the other pathways were remained.

### Selection of hub genes and molecular docking simulation

Targets that were involved in the above qualified pathways were selected out and scored further by the following scoring function:

$$ Norm(X)=\left(1-0.1\right)\ast \frac{X_i-\mathit{\operatorname{MIN}}(X)}{\mathit{\operatorname{MAX}}(X)-\mathit{\operatorname{MIN}}(X)}+0.1 $$

$$ {TS}_{\boldsymbol{j}}={N}_p\ast Norm\left(\sum \limits_{i=1}^{\boldsymbol{i}={N}_{\boldsymbol{c}}}{X}_i\right) $$

in which, *TS*_*j*_ refers to the score of the j-th target; *N*_*p*_ refers to the number of pathways that the j-th target involved in; *N*_*c*_ refers to the number of components that have interactions with the j-th target; *X*_*i*_ refers to the weight of the i-th component; *Norm* refers to the min-max normalization method with normalize data from 0.1 to 1.

After all targets were scored, targets that meet “score > 1.5 median score” were regarded as hub genes, and CTIs that were associated with these targets were selected out. The molecular docking simulation was further performed to detect whether the components and predicted targets form reasonable interactions and estimate their binding affinities.

First, we collected the crystal structures of the selected protein from the RCSB Protein Data Bank (PDB, http://www.pdb.org/, updated on 2020-4-10) and selected the relatively higher resolution crystal structures with the ligands. Second, the structures of chemical components contained in *A. euchroma* were downloaded from the NCBI PubChem Compound Database. Then, protein preparation module of Schrödinger’s Maestro Molecular modeling suite (Schrödinger Release 2015–2) was utilized for preparation of the protein crystallographic structures. Water molecules were subsequently deleted from the structures, and the amide moieties in the side chain were adjusted to optimize their interactions with surrounding residues and groups of atoms. Force field OPLS_2005 was also added. A ligand grid generation was based on the ligand in the co-crystallographic structure. The docking region was centered on the ligand, and after the ligand grid was generated, the compounds were imported into Mastero. LigPrep module of the Maestro molecular modeling package was used to obtain the 3D structures and energy minimization of the identified compounds. Compounds were docked in the generated grid using the standard Glide docking mode.

## Results

**Fig. 1:**
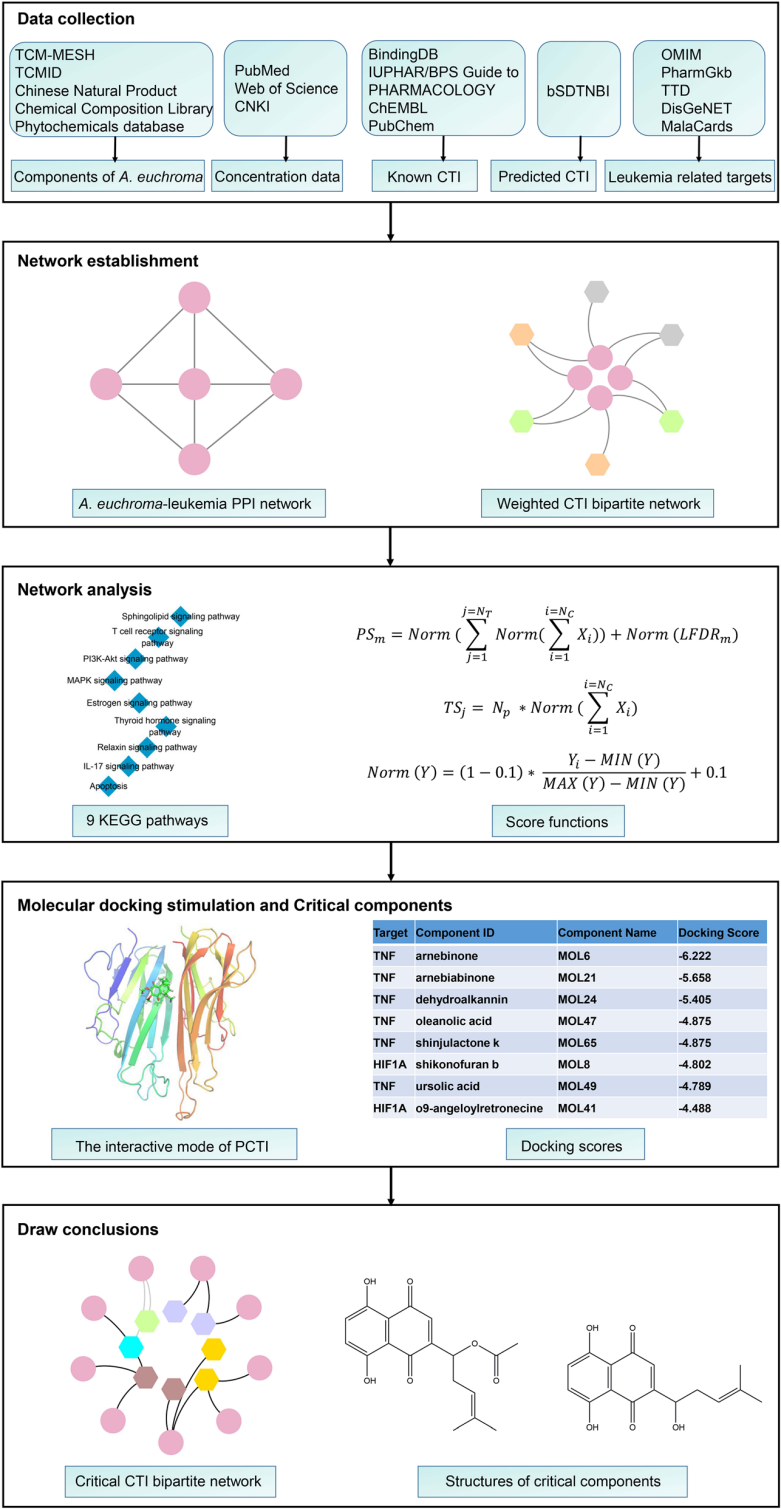
Workflow for *A. euchroma* on leukemia

In this study, we took the concentrations of components in *A. euchroma* into account, and analyzed the MoA of *A. euchroma* on leukemia by network pharmacology approach, which involved three steps in the whole workflow (Fig. 1): (1) data collection. The components of *A. euchroma* along with their concentrations, corresponding targets and leukemia related targets were identified by various databases and literature; the predicted targets were obtained through bSDTNBI method; (2) network establishment. *A. euchroma*-leukemia PPI network and weighted compound-target bipartite network were constructed through Cytoscape; (3) network analysis. Hub genes were selected out according to KEGG pathways analysis, and molecular docking was used to calculate whether the components from *A. euchroma* and predicted targets have interactions or not and to construct the critical compound-target bipartite network. Figure 1 depicts a workflow of the technical strategy used in this study.

### Components and concentrations of *A. euchroma* and leukemia related targets

During the process of data collection, all the components were collected from several databases and publications based on two criteria. On the one hand, the component should be found in at least two sources, such as two databases, or a database and a publication, or two publications; on the other hand, the component should have a clear structure. Finally, a total of 65 components of *A. euchroma* were obtained, and 27 of them with concentration information (see Supplementary Table S1). Considering that our bSDTNBI method could not distinguish stereoisomers, components that were mutually stereoisomers were treated as the same molecule among the 65 components.

According to the collected concentration information of each component, after the maximum and minimum values were removed, the average of percentage (mass percentage) was calculated. Finally, 11 components with concentration greater than 0.1% had a weight of 3 (Concentration Score, X = 3), 16 components with concentration less than 0.1% but greater than 0 had a weight of 2 (X = 2), and the remaining 38 components without collected concentration data had a weight of 1 (X = 1).

From OMIM, PharmGkb, TTD, DisGeNET, and MalaCards, a total of 332 targets were ultimately reserved after removing the duplicates.

### Known and predicted compound-target interactions

A total of 129 KCTIs for 13 components (MOL16, MOL18, MOL36, MOL38, MOL42, MOL43, MOL46, MOL47, MOL49, MOL53, MOL55, MOL56, and MOL57) were collected from databases, which were involved in 92 targets (see Supplementary Table S2). Only 13 of the 65 components of *A. euchroma* have known targets. Therefore, it is especially necessary to predict potential targets for components.

Herein, our bSDTNBI method was used to predict 10 targets for each of the 65 components. After grid search and 10-fold cross validation, the optimal values of the three parameters were set as α = 0.3, β = 0.1, γ = − 0.4. Then a total of 79 potential targets were obtained, resulting in 650 new CTIs. After the known and predicted CTIs were merged, duplicated CTIs were then removed. Finally, the number of retained CTIs was 779, involved in 65 components and 157 targets (see Supplementary Table S2).

### *A. euchroma*-leukemia PPI network

**Fig. 2:**
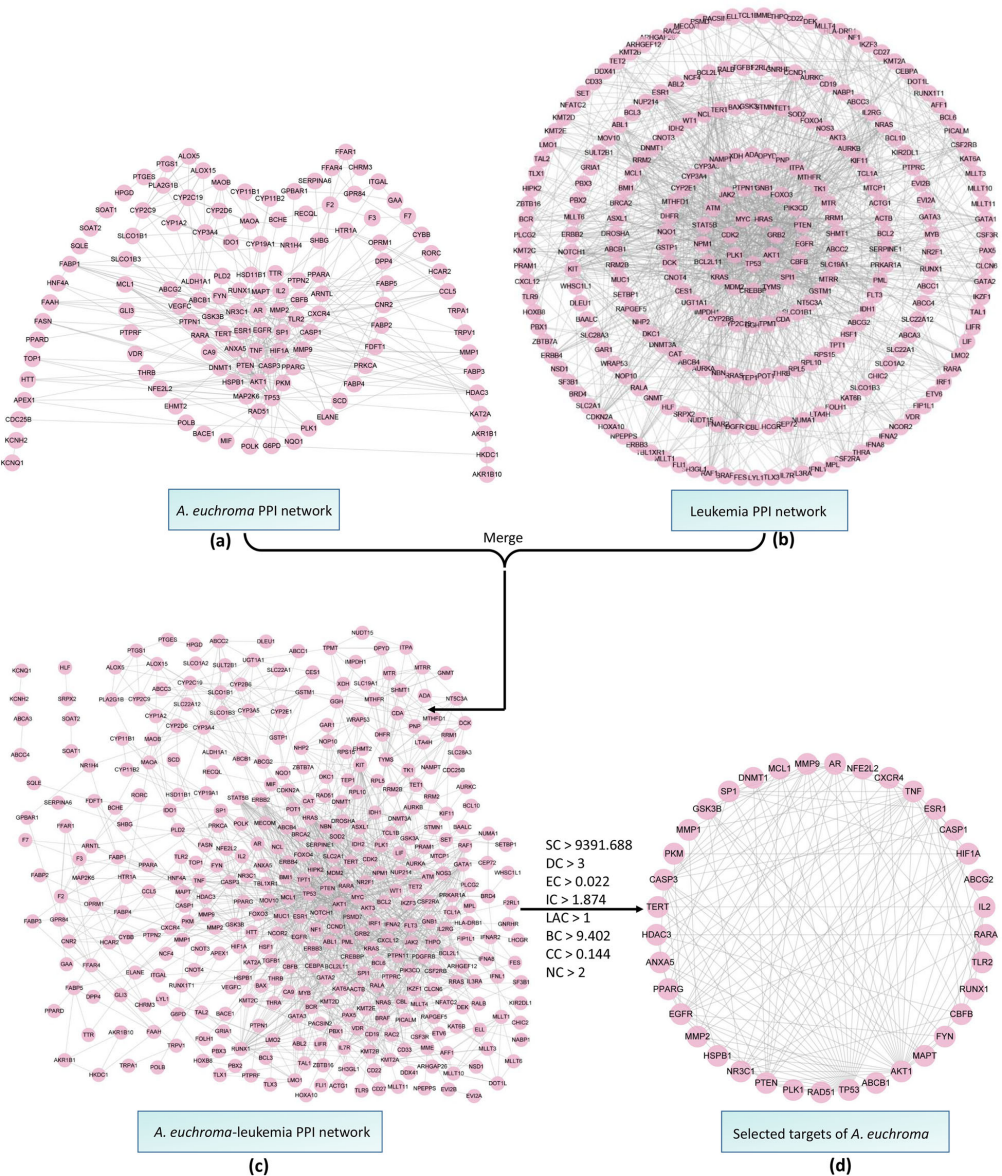
Construction of *A. euchroma*-leukemia PPI network. **a** *A. euchroma* PPI network (PPIA); **b** Leukemia PPI network (PPIL); **c** *A. euchroma*-leukemia PPI network; **d** Targets of *A. euchroma* that meet the screening criteria

157 targets (T_A_ = 157) of 65 components in *A. euchroma* and 332 leukemia related targets (T_L_ = 332) were imported to Cytoscape, respectively. When the confidence (score) cutoff was set to 0.7, 129 targets out of T_A_ had 364 PPIs (PPI_A_ = 364), while 270 targets out of T_L_ had 1335 PPIs (PPI_L_ = 1335). When they were merged, 1658 PPIs were obtained. Among these, there were 709 PPIs for the 129 targets of *A. euchroma* components. These 709 PPIs were selected out and re-submitted to Cytoscape to construct *A. euchroma*-leukemia PPI network. In the PPI network, eight central attributes were calculated by CytoNCA (see Supplementary Table S3). According to the screening criteria, there were 37 targets of *A. euchroma* components (T_AL_) were qualified (Fig. 2), which corresponding to 55 components and 114 CTIs.

### Weighted compound-target bipartite network

**Fig. 3:**
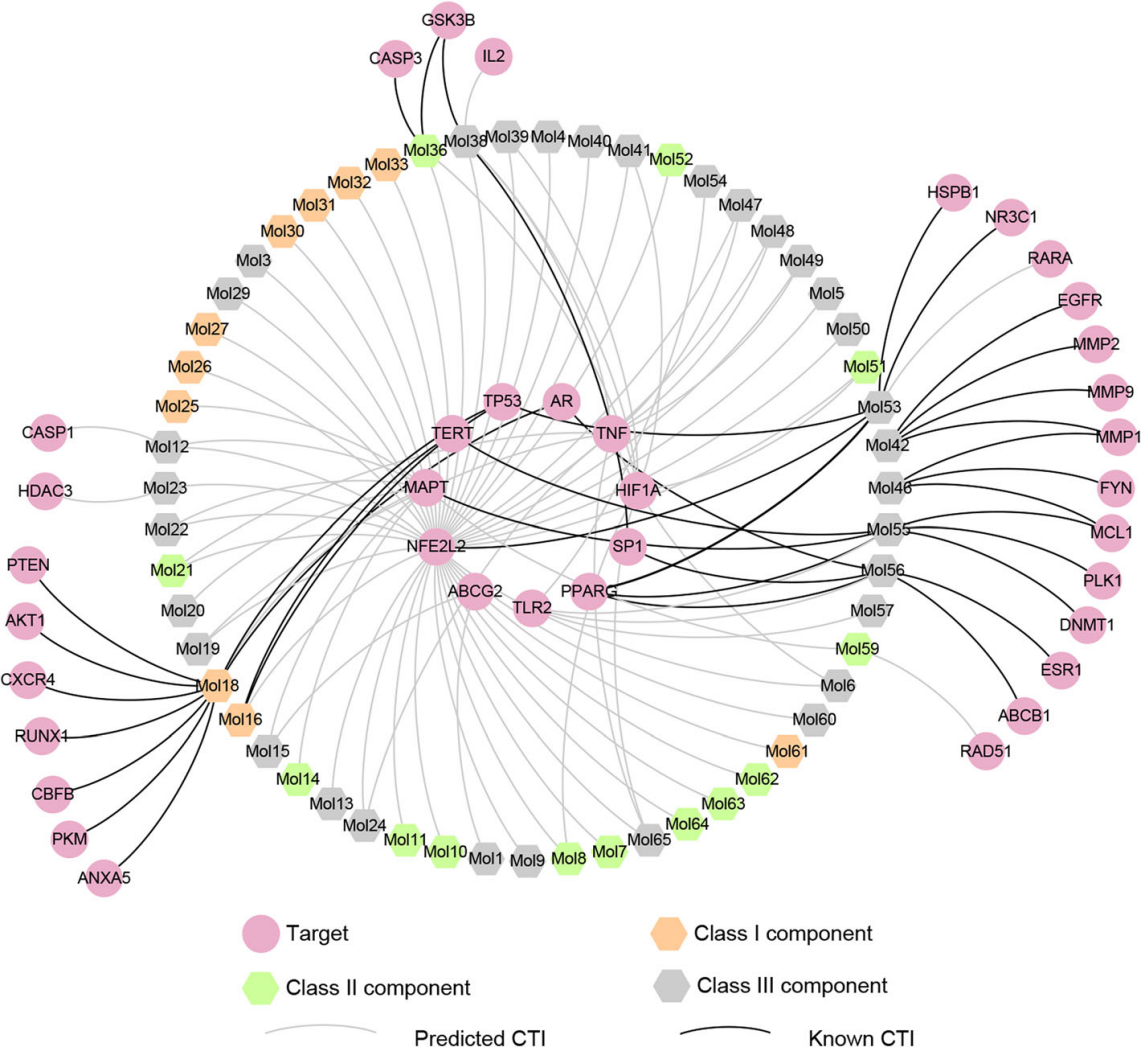
Weighted compound-target bipartite network. Pink circle nodes represent potential targets, hexagon nodes remark components and each edge represent the interaction between them. Orange hexagon nodes represent Class I components, which have the highest concentration; green hexagon nodes remark Class II components, which have a lower concentration; gray hexagon nodes remark Class III components, which have without concentration data. Silver edges represent predicted CTIs and black edges remark known CTIs

114 CTIs of the 37 T_AL_ were imported into Cytoscape to construct the weighted compound-target bipartite network, as shown in Fig. 3 (see Supplementary Table S4). Three weight values were given to each of the components according to their concentrations in *A. euchroma*. Figure 3 illustrated the network as a bipartite graph for the components and their potential targets with color-coded nodes. In total, this network consisted of 92 nodes and 114 edges, with 55 components as hexagon nodes and 37 targets as circle nodes.

To further clarify the relationships between the active compounds and their targets, we divided the 55 compounds into three groups according to their concentrations, namely Class I components, which have the highest concentration (orange node); Class II components, which have a lower concentration (green node); and Class III components, which do not have concentration information (gray node). There were 10 Class I components, 13 Class II components and 32 Class III components. Among Class I components, only MOL16 (acetylalkannin/acetylshikonin) and MOL18 (alkannin/shikonin) had interactions with more than one target; the remaining ones were associated with only one target. As for Class II components, eight components had interactions with one target, the others were associated with more than one target. NFE2L2 as the target with the most components, it could be hit by 42 components.

### Analysis of KEGG pathways

**Fig. 4:**
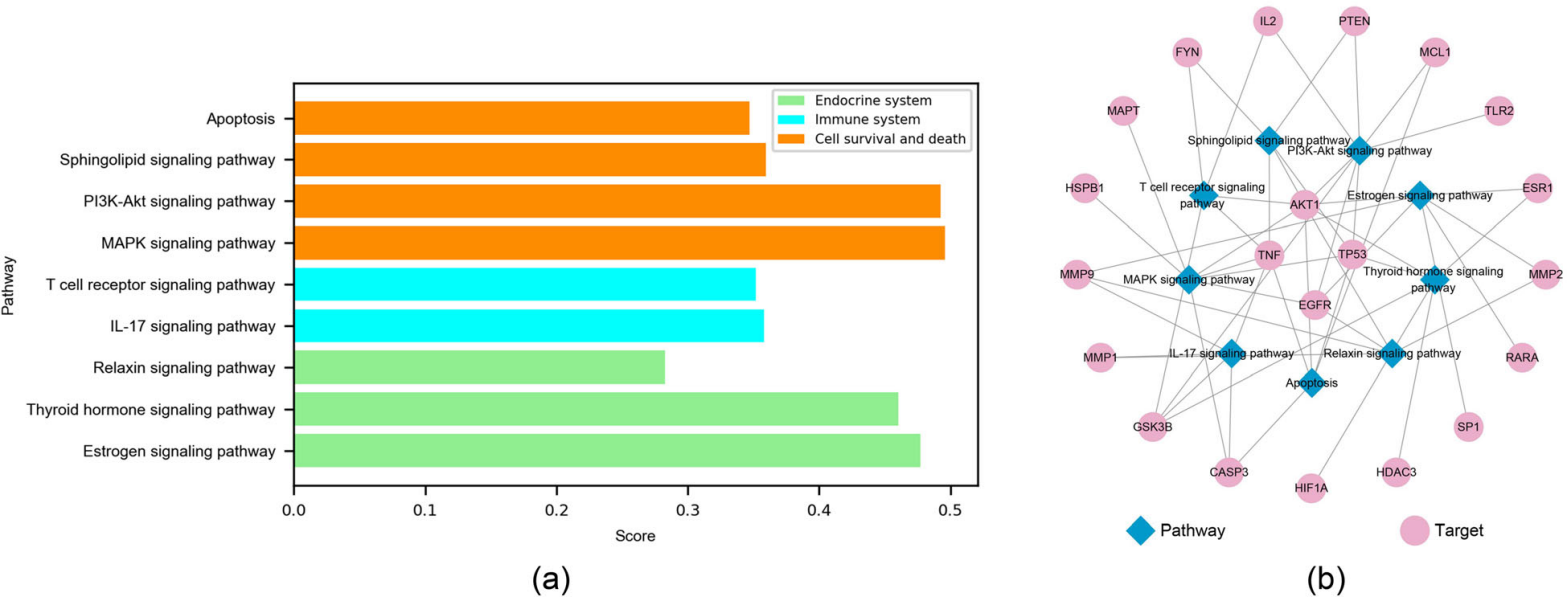
KEGG pathway analysis. **a** the score of 9 selected KEGG pathways. 9 pathways were divided into three categories, including four cell survival and death pathways (orange), two immune system pathways (cyan), three endocrine system pathways (green); **b** the selected 9 KEGG pathway-target interaction

37 T_AL_ were enriched into 115 KEGG pathways. According to the scoring function, 34 pathways were qualified. We divided the 34 pathways into four categories, including four cell survival and death pathways, two immune system pathways, three endocrine system pathways and 25 specific human diseases pathways (see Supplementary Table S5). For ease of analysis, we remove 25 specific human diseases pathways that are not directly related to possible leukemia treatment mechanisms, and retain other nine pathways. We constructed a pathway-gene network based on the nine pathway-related genes by Cytoscape to identify hub genes in the pathway. The pathway-gene network contained 30 nodes and 53 edges, including 21 genes and nine pathways (Fig. 4).

There were four cell survival and death pathways include MAPK signaling pathway (hsa04010), PI3K-Akt signaling pathway (hsa04151), Sphingolipid signaling pathway (hsa04071), Apoptosis (hsa04210). These four pathways were related to cell growth, differentiation and apoptosis. MAPK signal transduction pathway plays a crucial role in cell proliferation, differentiation and other processes. As a caspase activator, MAPK also plays an important role on apoptosis. AKT1, CASP3, EGFR, HSPB1, MAPT, TNF, and TP53 participated in the MAPK signaling pathway, and 22 components could act on them. PI3K/Akt signaling pathway is extensively present in cells. It is believed to be one of the pivotal signaling pathways regulating cell growth, proliferation, differentiation and apoptosis. Akt (gene symbol as AKT1) is a direct downstream target of PI3K/Akt pathway. Activated Akt can inhibit the release of cytochrome c from the mitochondria, which blockade the apoptosis triggered by caspases. The PI3K/Akt signaling pathway enriched eight genes, including AKT1, EGFR, GSK3B, IL2, MCL1, PTEN, TLR2, TP53, and a total of 11 components could act on them. Apoptosis is also known as programmed cell death, which plays a critical role in maintaining development, homeostasis and defending against pathogens. In transmitting apoptotic signals, CASP3 is the major effect factor. The activation of CASP3 triggers apoptosis. MCL1 is a member of the Bcl-2 family, which is an apoptotic inhibitor. Inactivation of p53 functions also causes cancer cells to escape apoptosis. The p53 plays a pivotal role in leukemic hematopoiesis. A total of five genes were enriched in Apoptosis signaling pathway, they were AKT1, CASP3, MCL1, TNF, TP53, and 15 components were associated with them. Analysis of the above results indicates that induction of cell apoptosis may be a key factor in the treatment of leukemia by *A. euchroma*.

There were two immune system pathways, include IL-17 signaling pathway (hsa04657) and T cell receptor signaling pathway (hsa04660). The IL-17 signaling pathway were enriched by five genes, include CASP3, GSK3B, MMP1, MMP9 and TNF. As for T cell receptor signaling pathway (hsa04660), AKT1, FYN, GSK3B, IL2 and TNF were enriched. It’s well known that cytokines play a critical role in the context of inflammation, and extensive research has also proved that cytokines exert profound effects on the progression of hematopoietic malignancies including leukemia. It can be inferred that *A. euchroma* relieves inflammatory environment may be related to its treatment of leukemia.

The other three pathways were endocrine system-related pathways, include Estrogen signaling pathway (hsa04915), Thyroid hormone signaling pathway (hsa04919) and Relaxin signaling pathway (hsa04926). A total of 12 genes enriched by these three pathways, they were AKT1, EGFR, ESR1, GSK3B, HDAC3, HIF1A, MMP1, MMP2, MMP9, RARA, SP1 and TP53. Of which, EGFR, one of the transmembrane tyrosine kinase receptors, serves as a stimulus for cancer growth, which is aberrantly activated in most common tumors, including leukemia.

### Molecular docking analysis and critical compound-target bipartite network

According to the scoring function, 10 hub targets (AKT1, CASP3, EGFR, GSK3B, HIF1A, MMP2, MMP9, PTEN, TNF and TP53) were qualified, and 18 components formed interactions with them in all CTIs. These components include arnebinone (MOL6), shikonofuran b (MOL8), acetylalkannin/acetylshikonin (MOL16), alkannin/shikonin (MOL18), arnebiabinone (MOL21), dehydroalkannin (MOL24), oleanolic acid (MOL47), shinjulactone k (MOL65), ursolic acid (MOL49), o9-angeloylretronecine (MOL41), caffeic acid (MOL42), linoleic acid (MOL53), daucosterol (MOL38), beta-sitosterol (MOL36), echimidine (Mol39), alkannin angelate (Mol19), 3-hydroxy-3-methyl butyric acid (Mol51), and tormentic acid (Mol48).

There were 27 CTIs between the 18 components and 10 targets, and 11 of them were KCTIs, 16 were PCTIs. All of the 16 PCTIs were composed of CTIs related to HIF1A and TNF while all of the CTIs related to HIF1A and TNF were PCTIs. In this study, we used the molecular docking stimulation to identify the binding ability of the 16 pairs of PCTIs that between components of *A. euchroma* and the obtained hub genes.

**Fig. 5:**
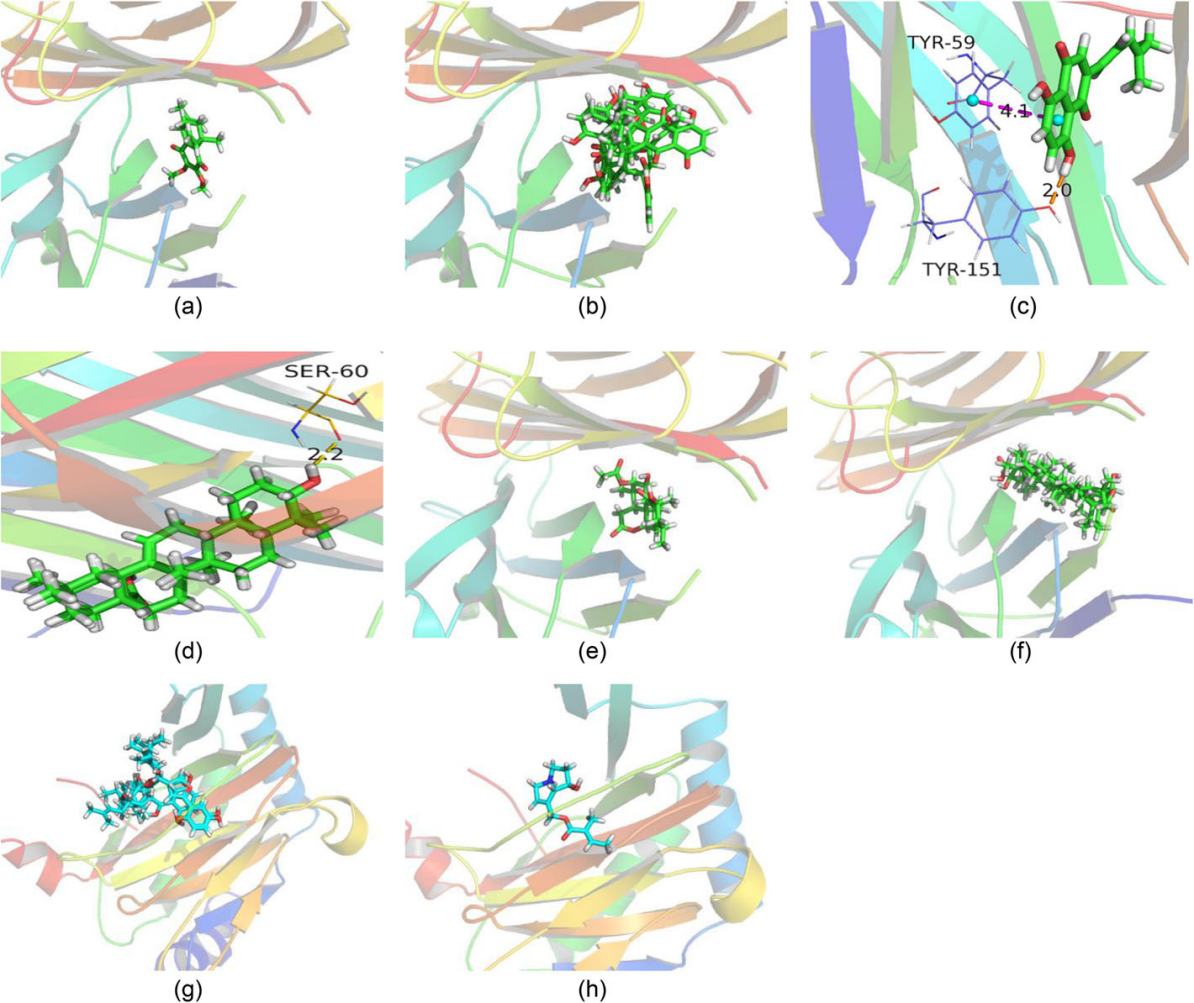
The interactive modes of 2 targets and the corresponding 8 compounds. **a** arnebinone to TNF (docking score = − 6.222); **b** arnebiabinone to TNF (docking score = − 5.658); **c** dehydroalkannin to TNF (docking score = − 5.405); **d** oleanolic acid to TNF (docking score = − 4.875); **e** shinjulactone k to TNF (docking score = − 4.875); **f** ursolic acid to TNF (docking score = − 4.789); **g** shikonofuran b to HIF1A (docking score = − 4.802); **h** o9-angeloylretronecine to HIF1A (docking score = − 4.488)

As the above described, only HIF1A and TNF-related CTIs were predicted, the other hub genes and their associated CTIs were KCTIs. Therefore, the molecular docking simulation was further performed to determine the binding level between the two predicted targets (HIF1A and TNF) and their corresponding components containing in *A. euchroma*. The docking scores of 16 pairs of PCTIs were listed in Table S6. There were eight pairs of PCTIs had strong binding free energy, since their docking scores were higher than the median value of all pairs. The eight pairs of PCTIs consisted of six pairs of TNF-related PCTIs and two of HIF1A. The action modes between TNF and its corresponding compounds were shown in Fig.5a-f, the interplay between HIF1A and its corresponding compounds were shown in Fig.5g-h. It can be found that dehydroalkannin (MOL24) could form a hydrogen bond with Tyr151, oleanolic acid (MOL47) could form a hydrogen bond with Ser60. In addition, dehydroalkannin (MOL24) could form a π-π interaction with Tyr59. Besides, arnebinone (MOL6) had the best binding ability with TNF (docking score = − 6.222), and followed by arnebiabinone (MOL21) (docking score = − 5.658), dehydroalkannin (MOL24) (docking score = − 5.405).

**Fig. 6:**
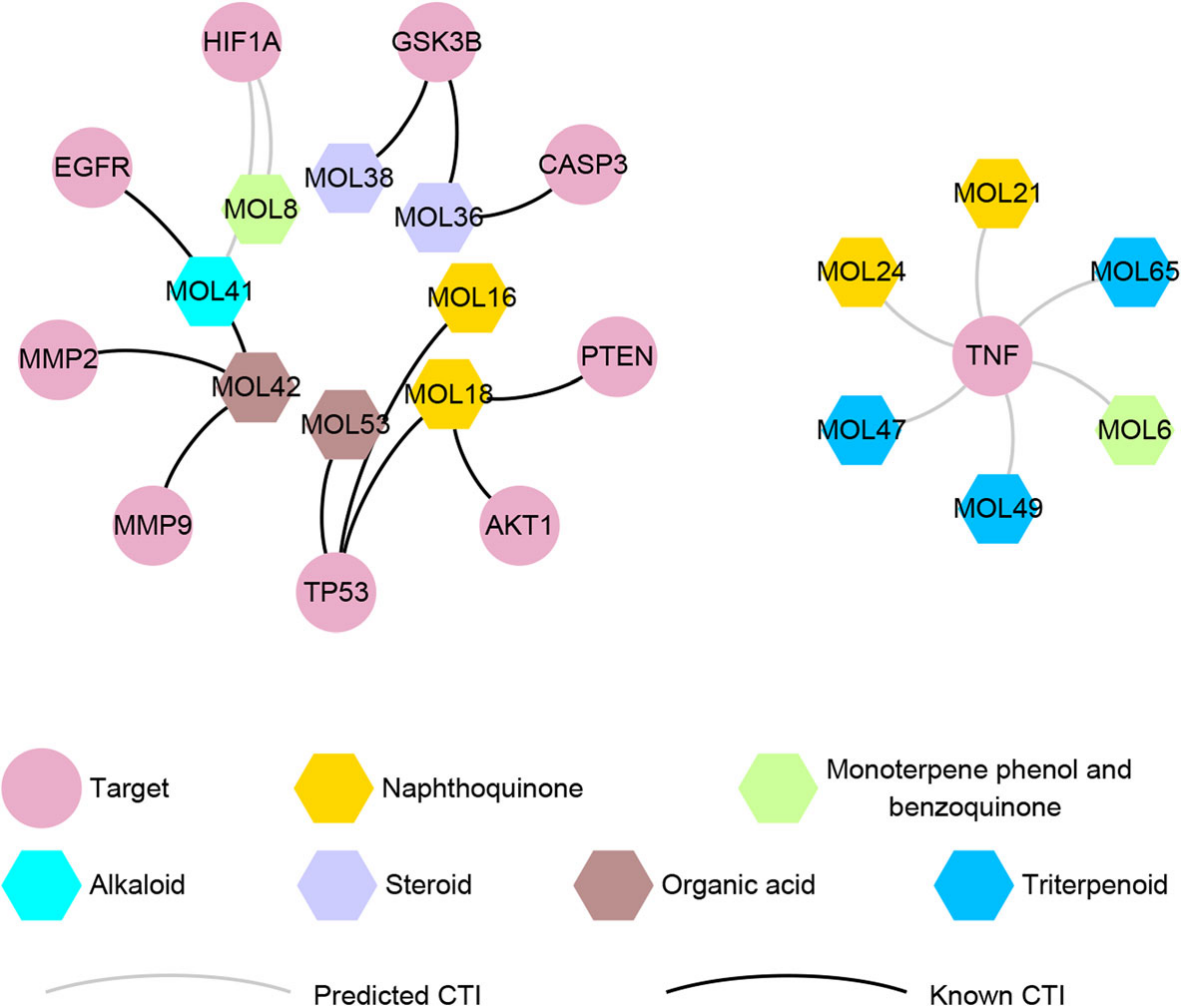
Critical compound-target bipartite network. Pink circle nodes represent critical targets, hexagon nodes remark components and each edge represent the interaction between them. Orange nodes represent naphthoquinones; green nodes remark monoterpene phenol and benzoquinones; cyan nodes remark alkaloids; purple nodes represent steroids; brown nodes remark organic acids; blue nodes represent triterpenoids. Silver edges represent predicted CTIs and black edges remark known CTIs

The eight pairs of PCTIs and 11 pairs of KCTIs were used to critical compound-target bipartite network, which involved in 10 targets and 14 components, as shown in Fig. 6. Here, two (MOL6 and MOL8) components were monoterpene phenol and benzoquinones **(**green nodes**)**, four (MOL24, MOL21, MOL18 and MOL16) were naphthoquinones **(**gold node**)**, three (MOL65, MOL47 and MOL49) were triterpenoids **(**blue nodes**)**, one (MOL41) was alkaloids **(**cyan node**)**, two (MOL38 and MOL36) were steroids **(**purple nodes**)**, and the other two (MOL42 and MOL53) were organic acids **(**brown nodes**)**. Structures of the 14 components were shown in Supplementary Figure S2.

## Discussion

*A. euchroma* has been used to treat blood diseases, including leukemia in the clinical of Chinese medicine [3]. In recent years, more and more studies have indicated that shikonin and some of its derivatives can inhibit leukemia cell proliferation, induce leukemia cell differentiation and promote apoptosis [9–11]. Most of the studies focus on individual or several ingredients, and the effects of all components of *A. euchroma* have never been explored systematically. Network pharmacology focuses on “multi-constituents, multi-targets to treat diseases”, which coincides with the holistic and systematic concepts of TCM [33]. Moreover, although the importance of TCM dosage has received widespread attention, few TCM-related network pharmacology studies have considered the influence of ingredient concentration on the effectiveness of TCM. In this study, we employed network pharmacology approach to explore the MoA of *A. euchroma* on leukemia. We retrieved 65 components and 157 targets of *A. euchroma* from known databases and literature. Combined with network analysis and molecular docking simulation, we found that 14 components and 10 targets were potential critical components and targets, which might contribute to the effect of *A. euchroma* on leukemia treatment. The 14 components include alkannin/shikonin (MOL18), arnebinone (MOL6), shikonofuran b (MOL8), arnebiabinone (MOL21), acetylalkannin/acetylshikonin (MOL16), dehydroalkannin (MOL24), oleanolic acid (MOL47), shinjulactone k (MOL65), ursolic acid (MOL49), o9-angeloylretronecine (MOL41), caffeic acid (MOL42), linoleic acid (MOL53), daucosterol (MOL38), beta-sitosterol (MOL36). The 10 targets include AKT1, CASP3, EGFR, GSK3B, HIF1A, MMP2, MMP9, PTEN, TNF and TP53. Our results indicated that the possible MoA might be involved in three aspects: 1. *A. euchroma* inhibits leukemia cell survival and induces apoptosis; 2. *A. euchroma* relieves inflammatory environment; 3. *A. euchroma* inhibits angiogenesis.

Apoptosis, also known as programmed cell death, is a physiological process or some pathological condition, in which the cells take part in the death process after they are triggered by a certain signal. Inhibiting leukemia cell survival and inducing leukemia cell apoptosis is a promising therapeutic approach for leukemia. There were four pathways (MAPK signaling pathway (hsa04010), PI3K-Akt signaling pathway (hsa04151), Sphingolipid signaling pathway (hsa04071), Apoptosis (hsa04210)) were related to cell survival and apoptosis, and 13 targets, 26 components in weighted compound-target bipartite network were mapped onto the four pathways. When transmitting apoptotic signals, CASP3 is the major effect factor. The activation of CASP3 triggers apoptosis. As one of the caspase activators, MAPK is well known to play a crucial role in apoptosis. Extensive studies have shown that targeting to MAPK signaling cascades, alone or in combination with other drugs, results in enhanced anti-leukemic responses in AML [34, 35]. It has been shown that activation of p38 MAPK promoted BCL2 degradation, which partly induced K562 cell apoptosis [36]. Huang et al. reported that shikonin treatment activated p38 MAPK and JNK phosphorylation in human lens epithelial cells [37]. The PI3K/Akt signaling pathway regulates diverse cellular processes, including growth, proliferation, differentiation and apoptosis by phosphorylating its downstream target, including BCL2L11, BAD, MDM2, XIAP, CASP9, GSK3B and so on. TP53 is a tumor suppressor protein and regulator of the cell cycle, which plays a pivotal role in leukemic hematopoiesis, but its functions are frequently suppressed by MDM2. Therefore, inhibition of MDM2 is beneficial to the stability of TP53. However, Akt enhances MDM2 activity, which is harmful for the stability of TP53. PTEN is a main negative regulator of the PI3K/Akt pathway, and plays an important role in maintaining hematopoietic stem cells and preventing leukemia [38]. It has been demonstrated that impaired PTEN function and aberrantly activated PI3K/Akt were present in many leukemia cases [9, 39]. Pan et al. suggested that combined BCL2 inhibition and TP53 activation may be a promising therapeutic approach for AML [40].

It is well known that inflammation is one of the major barriers to cancer therapy. In recent years, it has become evident that inflammation plays a key role in leukemia [41]. There were two pathways, including IL-17 signaling pathway (hsa04657) and T cell receptor signaling pathway (hsa04660) were related to inflammation. A total of eight targets were enriched into inflammation-related pathway, they were TNF, IL2, CASP3, GSK3B, MMP1, MMP9, AKT1 and FYN. And these eight targets had interactions with 13 components in the weighted compound-target bipartite network. As a pro-inflammatory cytokine, TNF is recognized as a key mediator of inflammatory reactions in tumor tissues, and also responsible for increased NF-κB activity in many tumors. TNF promotes the progression of several hematopoietic tumors by participating in several signaling pathways, including NF-κB, and PI3K/Akt [42, 43]. It has been reported that TNF can be produced by various leukemia cells, including AML [34], ALL [44], CML [45], CLL [46], and so on. There was also evidence showing that TNF induced MMP9 expression or secretion in leukemia cells [47]. TNF has been used as an anti-cancer drugs in various cancer cells. Staniforth et al. found that shikonin (MOL18), isobutyrylshikonin (MOL26), acetylshikonin (MOL16), β, β-dimethylacrylshikonin (MOL31) and isovalerylshikonin (MOL27) showed significant dose-dependent inhibition of TNF in vivo [48].

Angiogenesis is a critical element to cancer cell survival, and increased vascularity was found in patients with AML [49]. HIF1A, a key regulator of the cellular response to hypoxia, controls a vast array of gene products involved in energy metabolism, glycolysis, angiogenesis, apoptosis and cell cycle [50]. HIF1A has been recognized as a strong promoter of tumor growth and it is responsible for VEGF gene expression [51]. VEGF is one of the major angiogenesis-activating protein, it has been implicated in leukemia-associated angiogenesis [52]. There were some data showed that HIF1A over-expressed in some leukemia cells [53, 54]. Frolova et al. reported that blockade of HIF1A-mediated signaling may enhance the efficacy of the therapeutic regiments in ALL [55]. As one of the important factors to promote angiogenesis, matrix metalloproteinase (MMPs) were found highly expressed in B-CLL cells, and promoted the migration and invasion of leukemia cells [56]. MMP9, secreted by leukemic cells, mediates opening of the blood–brain barrier by disrupting tight junction proteins in CNS leukemia [57]. Sustained or enhanced MMP9 secretion plays an important role in the pathophysiology of tumor progression.

On the basis of the targets of *A. euchroma* discovered by network pharmacology, molecular docking simulation was used to explore the binding ability of between components and proteins. This technique is structure-based and could help researchers discover the interactions between the components of TCM and network targets. In this study, HIF1A and TNF were selected for molecular docking studies because they were predicted targets for components. TNF was involved in five pathways, include IL-17 signaling pathway (hsa04657), MAPK signaling pathway (hsa04010), T cell receptor signaling pathway (hsa04660), Sphingolipid signaling pathway (hsa04071) and Apoptosis (hsa04210), which were related to both apoptosis and inflammation. The molecular docking results indicated that six components (MOL6, MOL21, MOL24, MOL47, MOL65, and MOL49) had strong binding free energy with TNF, and two components (MOL8 and MOL41) had good binding affinity with HIF1A, since their docking scores were higher than the median value of all pairs. Among the eight components, MOL6 and MOL8 were monoterpene phenol and benzoquinones, MOL24 and MOL21 were naphthoquinones, which were the two main types of active components in *A. euchroma*.

According to the above analysis, we selected 10 targets and 14 components as critical targets and key components. Among these, shikonin (MOL18) was the most studied components of *A. euchroma*, it was demonstrated to have anti-inflammatory and pro-apoptotic effects [10, 58]. Acetylshikonin (MOL16) was found to induce tumor cell apoptosis through activating the pro-apoptotic bcl-2 family and caspase-3 [59].

As one of the most famous Chinese medicine for treating leukemia, ATO is an effective and relatively safe drug in the treatment of APL (acute promyelocytic leukemia, a subtype of AML) [2]. Combining compound-target network of *A. euchroma* and related literature, we compared ATO and *A. euchroma* in terms of leukemia treatment, and we found some commonalities: (1) MAPK signaling pathway. ATO has been demonstrated to activate all three MAPKs pathways, ERKs, JNKs, and p38 kinases to exert its anti-leukemia effect [60]. The MAPK signaling pathway was also included in pathways that enriched by targets of *A. euchroma*. (2) Angiogenesis. ATO was reported to inhibit angiogenesis by inhibiting the production of VEGF in leukemic cell [61]. As for *A. euchroma*, seven components could act on HIF1A, which is responsible for increased expression of VEGF. Moreover, several studies have shown that some components of *A. euchroma* exhibited antiangiogenic activity by inhibiting VEGF [62, 63]. (3) Caspases. ATO was shown to increase caspases activity to induce apoptosis of leukemia cells [64]. Extensive studies have demonstrated that components of *A. euchroma* increased the expression of caspases to induce apoptosis [10, 65]. As a result, we have reasons to speculate that *A. euchroma* may be a potential anti-leukemia agent with less toxicity than ATO.

In this study, we found that the MoA of *A. euchroma* on leukemia involves the inhibition of leukemia cell survival and induction of leukemia cell apoptosis, the relief of inflammatory environment, and the inhibition of angiogenesis. Concretely speaking, it includes three aspects: (1) *A. euchroma* impacts the PI3K/AKT and MAPK signaling pathway, which inhibit leukemia cell survival and induce leukemia cell apoptosis. (2) *A. euchroma* inhibits the expression of inflammatory medium, such as TNF, IL6, IL2 and so on to relieve inflammatory environment. (3) *A. euchroma* inhibits angiogenesis by regulating HIF1A, MMP2, MMP9 and other factors related to angiogenesis. The results preliminarily validated and explained the therapeutic material basis and mechanism of *A. euchroma* on leukemia, which provided a preliminary information and basis for further in-depth exploration of its MoA, and a reference for the study of the more complex components of TCM.

## Conclusions

Considering the concentration information of components in *A. euchroma* and combined with the methods of network analysis and molecular docking simulation, we found that 55 active components and 37 targets might be related to the anti-leukemia effects of *A. euchroma*, of which 14 components (MOL6, MOL8, MOL16, MOL18, MOL21, MOL24, MOL47, MOL65, MOL49, MOL41, MOL42, MOL53, MOL38, and MOL36) and 10 targets (AKT1, CASP3, EGFR, GSK3B, HIF1A, MMP2, MMP9, PTEN, TNF, and TP53) were considered to be critical. It was demonstrated that *A. euchroma* has potential anti-leukemia activities because of its effects on inhibiting leukemia cell survival and inducing apoptosis, relieving inflammatory environment and inhibiting angiogenesis. We further realized that, not only shikonin, the most fully studied ingredient of *A. euchroma*, one of compound of naphthoquinones, other components among the 14 critical components of *A. euchroma*, including monoterpene phenol and benzoquinones, triterpenoids, alkaloids, steroids and organic acids would also have therapeutic effects on leukemia.

## Supplementary information

**Additional file 1.** Figure S1. Two configurations of naphthoquinones.

**Additional file 2.** Figure S2. Structures of the 14 critical components.

**Additional file 3 **Table S1. Components of *A. euchroma* and content score.

**Additional file 4.** Table S2. Known and predicted component-target interactions.

**Additional file 5.** Table S3. Eight central attributes of targets.

**Additional file 6.** Table S4. Weighted component-important target interactions.

**Additional file 7.** Table S5. 34 KEGG pathways.

**Additional file 8.** Table S6. Docking scores of 16 pairs of PCTIs.

## Data Availability

The data can be requested from the author.

## Abbreviations

*A. euchroma*: *Arnebia euchroma*
ALL: Acute lymphoblastic leukemia
AML: Acute myeloid leukemia
APL: Acute promyelocytic leukemia
ATO: Arsenic trioxide
BC: Betweenness centrality
bSDTNBI: balanced substructure-drug-target network-based inference
CC: Closeness centrality
CLL: Chronic lymphoblastic leukemia
CML: Chronic myeloid leukemia
CTIs: Compound-target interactions
DC: Degree centrality
EC: Eigenvector centrality
FDR: False Discovery Rate
IC: Information centrality
KCTIs: Known compound-target interactions
LAC: Local average connectivity-based method
MMPs: Matrix metalloproteinase
MoA: Mechanism of action
NBI: Network-based inference
NC: Network centrality
PCTIs: Predicted compound-target interactions
PDB: RCSB Protein Data Bank
PPI_A_: *A. euchroma* PPI network
PPI_L_: Leukemia PPI network
PPIs: Protein-protein interactions
SC: Subgraph centrality
SDTNBI: Substructure-drug-target network-based inference
T_A_: Targets of components of *A. euchroma*
T_AL_: Leukemia-related targets of *A. euchroma*
TCM: Traditional Chinese medicine
T_L_: Targets related to leukemia
